# Soil as a transdisciplinary research catalyst: from bioprospecting to biorespecting

**DOI:** 10.1098/rsos.230963

**Published:** 2023-11-15

**Authors:** Matthew J. Tarnowski, Gilda Varliero, Jim Scown, Emily Phelps, Thomas E. Gorochowski

**Affiliations:** ^1^ School of Biological Science, University of Bristol, 24 Tyndall Avenue, Bristol BS8 1TQ, UK; ^2^ School of Biosciences, Geography and Physics, Swansea University, Swansea SA2 8PP, UK; ^3^ Rhizosphere Processes Group, Swiss Federal Research Institute WSL, 8903 Birmensdorf, Switzerland; ^4^ Humanities and Social Sciences, University of Exeter, Cornwall TR10 9FE, UK; ^5^ BrisEngBio, University of Bristol, Cantock's Close, Bristol BS8 1TS, UK

**Keywords:** public engagement, metagenomics, transdisciplinary, metaphor, soil, synthetic biology

## Abstract

The vast microbial biodiversity of soils is beginning to be observed and understood by applying modern DNA sequencing techniques. However, ensuring this potentially valuable information is used in a fair and equitable way remains a challenge. Here, we present a public engagement project that explores this topic through collaborative research of soil microbiomes at six urban locations using nanopore-based DNA sequencing. The project brought together researchers from the disciplines of synthetic biology, environmental humanities and microbial ecology, as well as school students aged 14–16 years old, to gain a broader understanding of views on the use of data from the environment. Discussions led to the transformation of ‘bioprospecting’, a metaphor with extractive connotations which is often used to frame environmental DNA sequencing studies, towards a more collaborative approach—‘biorespecting’. This shift in terminology acknowledges that genetic information contained in soil arises as a result of entire ecosystems, including the people involved in its creation. Therefore, any use of sequence information should be accountable to the ecosystems from which it arose. As knowledge can arise from ecosystems and communities, science and technology should acknowledge this link and reciprocate with care and benefit-sharing to help improve the wellbeing of future generations.

## 1. Introduction

Soils are complex, alive and entwined with civilization [1]. Prehistoric soil practices are evident around the world [2,3] and are likely the consequence of millenia of research unbounded by scientific disciplines. More recently, the science of soils fractured into disciplines, a process that continues today, but which has its roots in the nineteenth century [4]. Charles Darwin studied soils from a biological perspective, investigating the role of worms in their formation [5], while the mid-nineteenth century German chemist Justus von Liebig forged a way to define soil as a chemical system [6]. Understanding soil in terms of its chemical properties has maintained popularity to this day: an analysis of biological, chemical and physical indicators included in soil-health assessment schemes between 1980 and 2020 showed that throughout this time period, chemical and physical indicators are most widely used [7]. Thus, while a multitude of disciplines research soils, each uses a set of different approaches to understand them in various ways—through different epistemologies. Epistemology is a branch of philosophy that asks how we know what we know [8]. The same soil, therefore, can be understood in many different ways, but what happens when these perspectives collide?

A biological perspective to soil management is urgently needed and ‘critical to expanding soil assessment and management to address concerns over biodiversity, water quality, climate recreation and human and planetary health’ [7]. Metagenomics is a modern approach for studying soil microbiology [9]. It enables the characterization of the genetics of the microorganisms living in a soil sample and many academic disciplines now use metagenomics as a central means to study the organisms present in soils. Synthetic biology, which aims to design and engineer living organisms with novel functionalities [10–12], typically uses metagenomic studies as a source of genetic parts [13,14]. To a synthetic biologist, genetic parts are DNA sequences that encode a self-contained biological component [15,16]. This could be a gene product that could be synthesized (e.g. a functional protein like an enzyme) or control elements that regulate the processes underlying gene expression (e.g. promoters that regulate transcription rates or ribosome binding sites that regulate translation rates) [17]. Often, a ‘bioprospecting’ metaphor motivates metagenomic studies with the idea that the researcher is searching the biodiversity of the soil for new genetic parts [13]. Language used during research can influence forms of inquiry across disciplines [18]. For example, ‘feedback metaphors’ contain cultural assumptions and therefore affect the values of scholarly work and how research proceeds—the metaphors drive the scholarship [19]. Feedback metaphors extend beyond scientific research, to encourage particular visions of the world [19], and can ‘become self-reinforcing prophecies' [20] that frame the non-human world as a pool of resources, with the potential for exploitation by humans.

The bioprospecting metaphor arises, at least in part, from histories of extractive industry, most notably mining. A broader history of the extractive epistemologies in the sciences has been traced, recognizing ‘global commodification of life through the exploitation of two non-commodities: labour and nature’ [21]. In this extractive mindset, knowledge is taken in a one-way transaction on the terms of the scientist without wider engagement and can lead to the exploitation of people, other living organisms and inorganic nature without care or reciprocity. It has also been argued that the extractive bioprospecting approach leads to biopiracy [22]. Biological material is regulated by the Nagoya Protocol on Access and Benefit-sharing [23]. However, it has been highlighted that reforms are needed in light of the digital sequence information (DSI) generated by DNA sequencing [24,25] and technical and legal challenges when applied to microbial ecology [26]. While best practice in bioprospecting has been proposed [27], alternative metaphors which are not rooted in extraction could change the future directions and impacts of soil DNA sequencing science and technology. The humanities have an awareness of the links of extractive epistemologies to the long and violent colonial histories of exploitation, affecting humans and other living organisms [28] and offer a good starting point to explore this idea.

The environmental humanities also provide further lenses through which soil can be studied. A range of scholarly approaches exist that understand environmental challenges as inextricable from social, cultural and human factors, including studying the imbrication of cultural, material and political ecologies [29]. The environmental humanities encompass a collection of disciplines and approaches, including environmental history, literary ecocriticism and environmental philosophy. Interdisciplinary by nature, environmental humanities scholarship examines relationships between the human and non-human and the ways that environments are understood by other disciplines, in particular, those in the environmental sciences. Recent work, drawing on postcolonial, as well as ecocritical approaches, has increasingly emphasized the often exploitative, extractive and uneven connections through which human and non-human lives (e.g. the animals, plants and microbes that share the environment) are entangled today and through the long histories of capitalism and colonialism [29]. A recent review presented an agenda for the collaboration of social sciences, humanities and natural sciences in microbiome research [30]. Thirty-six academics and stakeholders raised questions, which were organized into eight themes at the intersection of the microbiome and the following: health, lifestyle, environment, conceptualization, thinking, value, public communication and research [30].

**Figure 1.**
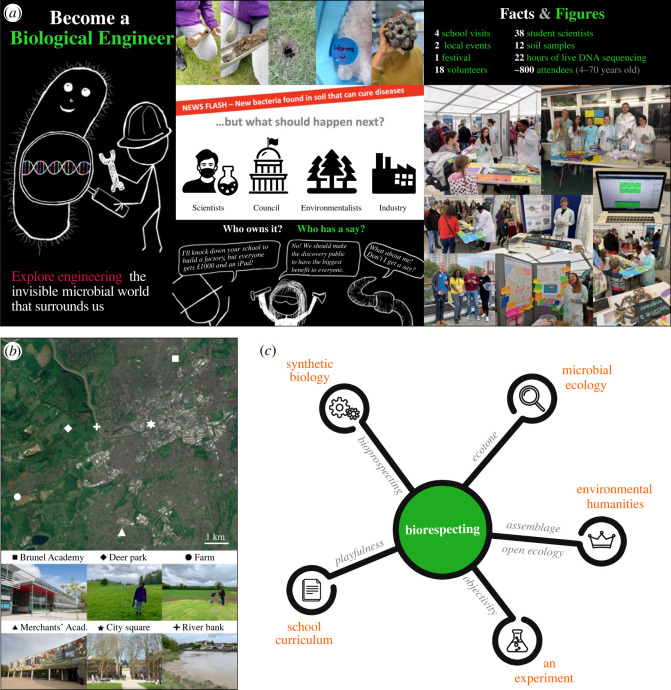
The Become a Biological Engineer (BaBE) public engagement project leads to transdisciplinary study of soil microbiomes. (*a*) Overview of the BaBE project. The project involved school visits to sample soil and debate what might happen if microbial DNA considered valuable was found (middle panel) and activities at local events and a festival (right panel). (*b*) Map of sample locations across the city of Bristol (UK) with representative pictures of the sample sites. (*c*) Schematic depicting the transdisciplinary research study. Researchers from different disciplines (orange) shared ideas (grey), leading to creation of a new perspective of ‘biorespecting’ (green) when considering soil research.

Here, we used soil as a catalyst for transdisciplinary collaboration exploring some of these ideas. The work centres around a public engagement project that explored the idea of using DNA sequencing of soil metagenomes as a source of new parts for engineering biology (figure 1*a*). Soil samples were taken from two schools, a riverbank, a public park, a research farm and a deer park (figure 1*b*). This was partly motivated due to a lack of studies of the microbiology of urban soils compared to the vast research carried out on natural and agricultural soils [31]. The project brought together a microbial ecologist, two synthetic biologists, an environmental humanities scholar and high-school students, as well as connecting more broadly with the general public through science festivals and community centre visits. Through these events, we debated the potential consequences of using DNA sequences generated by the soil metagenomics study in different ways and the long-term impacts and implications. These discussions caused a paradigm shift from the common perspective of ‘bioprospecting’, to what we term ‘biorespecting’ (figure 1*c*), which re-frames the traditional relationship between investigator and object of study as a collaboration between researcher and soil. Approaches that do not treat nature as a resource to be exploited deserve attention in science and technology as a whole. Collaborative approaches to science have wider relevance as they can potentially help shape what scientific research is done, how that research is conducted and the effects and implications of that research in the world. For this reason, we advocate for others to consider adopting the biorespecting concept moving forward, but doing so with an appreciation of the historical context of more exploitative perspectives like bioprospecting and the problems they can, and have, caused.

## 2. Results and discussion

### 2.1. Public engagement to support transdisciplinary research

The ‘Become a Biological Engineer’ (BaBE) public engagement project aimed to provide a space to learn about and question synthetic biology approaches to engineering biology and to better understand how biotechnology is perceived more widely. The project also attracted researchers from multiple disciplines (electronic supplementary material, table S2), motivated to engage with those outside of a university setting and better understand how their research was perceived (figure 1*a*). We focused the project on a technology widely used in synthetic biology—nanopore-based DNA sequencing, which involves reading DNA sequences as they pass through nanoscale protein pores [32]. Hands-on participatory activities were used to inspire people's imagination (Methods). Our post-activity questionnaire received 58 responses. Of these, 85% of participants felt they better understood how bioengineering worked, 71% thought it should be used for medicine or making sustainable materials and 45% wanted closer links to scientists when exploring new applications that might have unexpected outcomes.

We also organized two lessons for high-school students aged 14–16 years old, which aimed to be engaging and thought-provoking (Methods). In the first lesson (electronic supplementary material, note S1), we introduced the class to soil science, DNA sequencing, and how bioengineers can extract and make use of genes from organisms in natural environments (e.g. for the discovery and production of new antibiotics). Following this, the students were collectively allowed to decide where to sample soil from in their school to see what it contained and worked in groups to creatively describe the sample site (electronic supplementary material, table S4). These samples were then sequenced in the laboratory and live at the ‘Festival of Nature’ in Bristol.

In the second lesson (electronic supplementary material, note S2), we presented the sequencing results in comparison to other sites we had sampled across Bristol and asked the students to imagine that the experiment had uncovered a valuable new gene at their school that could support a revolutionary new medicine. The students were split into groups, with each being asked to play the role of an interested party and explain why their route forward should be taken (electronic supplementary material, note S3). Debates during the group activity were heated as questions around ownership and short- versus long-term benefits arose. At both schools, similar arguments were often raised for specific groups. For example, the benefit of large sums of money being provided by the multi-national company was highlighted as allowing people to move elsewhere and start a better life. But many students raised concerns about broken promises of large corporations they had seen in the news, a general lack of trust for large corporations who did not have their own interests at heart and were worried about what might happen to other people in the area and the wildlife living in the soil. Even so, many of the students felt strongly that some personal benefits were warranted as they had discovered this valuable resource. Therefore, making the medicine free to everyone as advocated for by the scientific community was deemed unfair. In all cases, it was clear to see the sudden realization of how scientific discoveries could potentially impact their personal lives and the dilemmas that arise around sharing the benefits of DNA sequences abundant in nature, but technically owned by no one. The session ended with a vote by the entire class for their preferred option. In all cases this led to desired outcomes that balanced exploitation of the newly found resource with long-term sustainability of the school and the local sampling site, which typically was a combination of the routes presented by all the groups—no single view was seen as the right way forward. Co-creating these lessons and the accompanying microbiome research led to an environment where transdisciplinary research could flourish.

We found that organizing, undertaking and reporting the public engagement activities (electronic supplementary material, table S5) provided a context that brought together researchers of different disciplines and stimulated deep discussions and the sharing of relevant ideas from their areas of expertise. While the outreach on its own did not lead to the transformation of the bioprospecting metaphor, the diverse environments and different forms of interaction it provided were a catalyst for beginning that process.

### 2.2. Analysis of urban soil microbiomes

**Figure 2.**
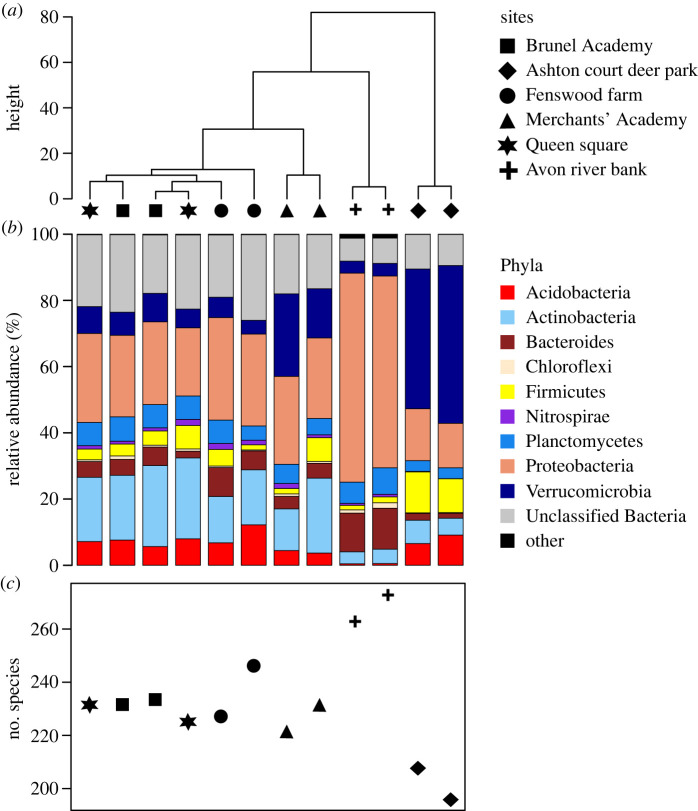
Analysis of soil samples from across the city of Bristol, UK. (*a*) Sample similarity clustering. (*b*) Microbial composition of the 12 soil samples at the phylum-level (only the most abundant phyla are shown, i.e. phyla with at least 1% of relative abundance in at least one sample). (*c*) Number of microbial species in each sample with greater than 1% relative abundance in at least one sample.

Environmental DNA analysis of soils from a range of environments across the city of Bristol resulted in between 104 846 and 198 062 sequences per sample with a mean read quality of ∼Q11 for all samples (electronic supplementary material, table S1 and figure S1). A quality score of Q11 corresponds to a probability to have one incorrect base in the DNA sequence every 20 bases which gives a good taxonomic resolution considering that the entire 16S rRNA gene was sequenced [33]. Analysis of the species present in the sample showed that samples collected from Ashton court deer park and Avon river bank formed two distinct clusters that differed from the other sampling locations. Interestingly, all samples from human-impacted sites (i.e. Merchant's Academy, Fenswood farm, Brunel Academy and Queen square) formed a cluster of more highly related communities (figure 2*a*). Compared to the human-impacted soils, microbial communities in the Avon river bank soil are characterized by a higher abundance of organisms belonging to Proteobacteria and Bacteroidetes. Whereas more organisms ascribed to the phyla Verrucomicrobia and Firmicutes were observed in the Ashton court deer park soil (figure 2*b*). The number of bacterial species observed in the different samples also followed a similar trend where the species number showed the highest values in the Avon river bank soils (263 and 269 species), the lowest values in the Ashton court deer park soils (194 and 201 species) and intermediate values in all the more human-impacted soils with values ranging between 225 and 245 species (figure 2*c*).

The differences observed in the Avon river bank and Ashton court deer park soils compared to the more human-impacted samples is not surprising. The soil sampled from the Avon river bank constitutes the most diverse microbial ecosystem among all the sampled sites, which is reflected in its diverse microbial composition. Furthermore, the Avon river bank in Bristol, being an ecotone between soil, freshwater and seawater systems, will tend to harbour more diverse bacterial communities compared to the other sampled soils [34]. In the case of the soils from the Ashton court deer park, animal grazing has previously been shown to impact soil microbial community structure and diversity [35–37]. The similar bacterial community structure and diversity observed in all the human-impacted soils, despite their different provenience (e.g. grass, crop field) and soil management strategies, could be explained by the similar degree of soil disturbance by human activities. In fact, soil disturbance can be an important driver to determine microbial communities in different environments such as semiarid grasslands, peatlands and brownfields [38–40]. The level of disturbance, urbanization and human density of a soil area is known to influence microbial community structure, diversity and function in the ecosystem [41–43]. All these aspects impact soil health and van Bruggen *et al.* [44] outline the concept that ecosystems have ‘one health’ connected by cycling of diverse microbial communities, particularly those of the soil.

**Figure 3.**
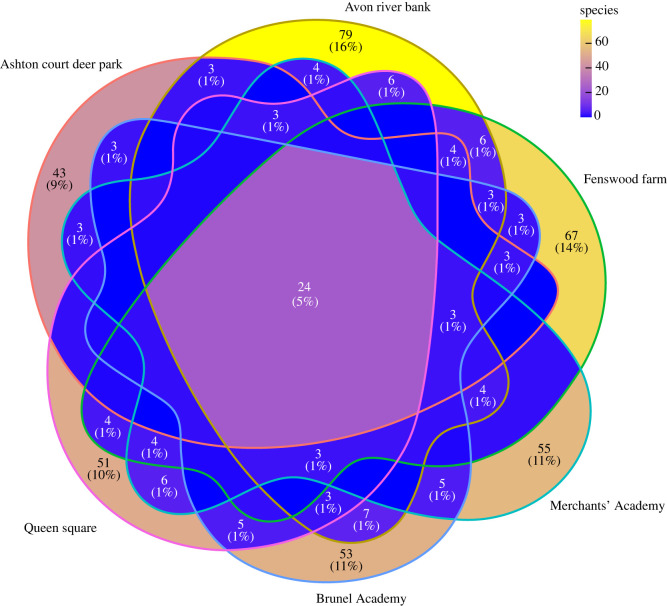
Number of shared species across different sampling sites. Only percentages greater than or equal to 1% are reported.

While differences were observed in the microbial composition and diversity of human-impacted soils versus the Avon river bank soil and Ashton court deer park soil (figure 2*a*,*b*), the samples from the human-impacted soils did not have more species in common between each other than with the other two sites (figure 3). This suggests that diverse human-impacted soils can harbour diverse microorganisms which are specific to unique soil conditions and environmental characteristics. In fact, further to human impact and disturbance, soil microbial communities are also influenced by several factors such as nutrient and water availability, vegetation cover and environmental connectivity [45–48], where microbial life conditions can also vary at the level of micro-environments [49].

**Figure 4.**
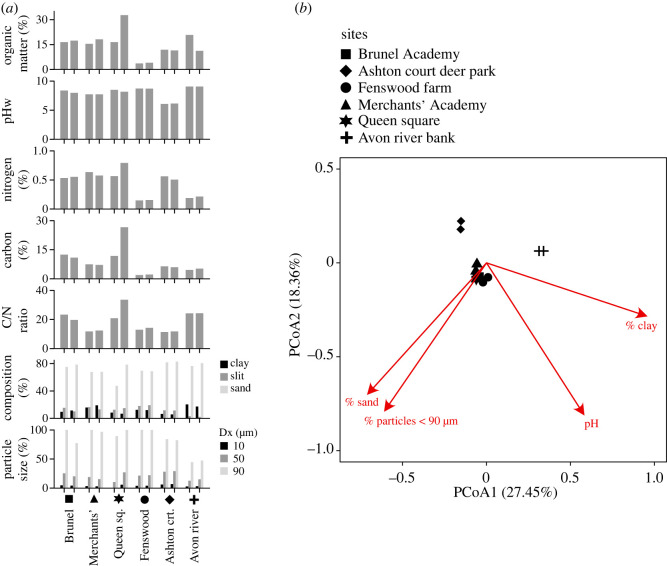
Overview of geochemical analysis. (*a*) Geochemical features measured across all soil samples. pHW: pH of the soil in water; C/N ratio: carbon to nitrogen ratio; Dx(10 µm): percentage of particles less than 10 µm in diameter, Dx(50 µm): percentage of particles less than 50 µm in diameter, Dx(90 µm): percentage of particles less than 90 µm in diameter. (*b*) Envfit analysis showing the key geochemical features across the soil samples (Methods). PCoA, principal coordinate analysis.

To assess whether microbial diversity might link to other features of the soils, we carried out geochemical analysis of all the soil samples and performed an ‘envfit’ analysis where we perform a multiple regression of environmental variable with ordination axes; the environmental variable is used as dependent and selected ordination axes as explanatory variables (Methods). From this, we found that microbial composition significantly correlated with the measured geochemical variables (*R* = 0.52, *p* = 0.005), but not with the geographical distances between samples (*R* = –0.27, *p* = 0.985). Geochemical variables that showed significant correlations in the envfit analysis were pH (*R*^2^ = 0.97, *p* = 0.001) and three variables linked to particle size: clay percentage (*R*^2^ = 0.52, *p* = 0.046), sand percentage (*R*^2^ = 0.75, *p* = 0.012) and size particle 90 µm (*R*^2^ = 0.67, *p* = 0.008) (figure 4, table 1). Both pH and soil particle composition have been indicated as major drivers of microbial distributions [50–53]. Furthermore, human activities are known to impact soil pH and particle size distributions [54,55], and thus consequently the microbial communities present.

**Table 1.** Envfit results.

geochemical feature	PCoA1^a^	PCoA2^a^	*R*^2^	*p*-value
organic matter	0.037	–0.999	0.007	0.954
pH	0.525	–0.851	0.985	0.001
% nitrogen	–0.965	0.263	0.396	0.105
% carbon	–0.672	–0.741	0.144	0.464
carbon/nitrogen ratio	0.628	–0.778	0.334	0.133
% clay	0.959	–0.280	0.443	0.068
% silt	0.330	0.944	0.313	0.180
% sand	–0.865	–0.501	0.772	0.007
% particles < 10 µm	–0.446	0.895	0.326	0.220
% particles < 50 µm	–0.789	0.614	0.399	0.103
% particles < 90 µm	–0.792	–0.610	0.682	0.006

^a^PCoA, principal coordinate analysis.

Although these results are limited by a lack of undisturbed soil with no human impact at all (e.g. soil from a woodland area that is protected and not subjected to grazing), they do show how microbial structure and diversity can potentially change among sites sampled within approximately 7 km^2^ and highlight how human-impacted soils differed from more unmanaged soils. Furthermore, a key outcome of these public engagement activities was to stimulate discussions between researchers (electronic supplementary material, table S2) in different disciplines that are often disconnected. In this case, between microbial ecology, synthetic biology and the environmental humanities. These discussions led us to explore how a range of epistemologies can be useful in fostering transdisciplinary research, something discussed further in the following sections.

### 2.3. Transdisciplinary approaches

Various discussions arising throughout the project brought forth approaches used in the environmental humanities that offered novel ways to understand both the research process and soils. For example, actor-network theory proposes that matter has agency and informs shifting assemblages of actors [56]. To complicate things further, actors can be human, non-human and non-individual [57]. This suggests that research is influenced by non-human and human actors, such as soil. In our study, the non-human actors include all the beings and forces that come together to form a particular soil, along with the researchers, technologies and disciplinary approaches that collaborate to understand soil as an object of research. Thus, human actors also go beyond the researchers to dog-walkers, other students, park rangers and so on. Soil research presents an opportunity for transdisciplinary research with a series of non-human actors that typify natural cultural relations [58].

Assemblage theory is another approach that researchers can use to understand the relation between their approaches to studying soil. An assemblage is ‘an arrangement or layout of heterogeneous elements' [59]. This is exemplified by our study, in which the soils under investigation can be understood as shifting assemblages of human and non-human actors, of which different disciplines (epistemologies) and the methods each use to study soil in different ways, are a part. Furthermore, social formations (such as disciplines) are assemblages of other complex configurations, such as soils, and they in turn play roles in other more extended configurations. Among other things, assemblage theory enables the idea of a shifting collection of disciplines, allowing us to come together without feeling fixed to unchangeable disciplinary dogmas. This perspective enables an openness whereby disciplines can change their approaches as a consequence of working together.

Though complex, these approaches can open fruitful ways of working across disciplines, be it multi-, inter- or trans-disciplinarily. Multidisciplinarity involves multiple disciplines working together. Interdisciplinarity involves a shared understanding of the different approach (epistemology) used by each discipline. Finally, transdisciplinary research allows space for disciplinary approaches to generate new knowledge of that subject which would not have been possible without the new disciplinary configuration. In our case, blending disciplinary approaches led to transformation of a disciplinary epistemology.

### 2.4. How can soil and research influence one another?

Interpreting soil research as a network of human and non-human actors led to the idea that soils and research practices influence one another. Returning to the concept of ‘one health’, van Bruggen *et al*. argue that ‘the health of all organisms in an ecosystem are interconnected and mediated through the cycling of subsets of microbial communities from the environment (in particular the soil) to plants, animals and humans, and back into the environment’ [44]. Health is defined as the absence of disease and the authors suggest that in the case of microbiomes, health arises from microbiome stability, resilience, diversity, connectedness and integrity of nutrient cycles and energy flows [44]. One health describes the connectedness of environmental microbiomes, connecting both soil and researchers at the level of microbiology.

The microbiomes we recovered reveal distinct collections, or ecologies, of microorganisms at each site. Similarly, the idea of ecologies of knowledge [60] emphasizes the plurality of knowledges, as exemplified by the diverse disciplines engaged in our study. Each discipline brings its own ecology of approaches, much like each microbiome brings an ecology of microbes. It was suggested that the relatively high diversity at the river bank arises from influxes of microorganisms from soil and water. Similarly, ecotones might be thought of in an epistemological sense as places where knowledges meet and which have unique properties, due to the diversity of ideas brought forward.

Ecologies, such as soils, have been described in the environmental humanities as ‘open’ rather than ‘closed’ [61]*.* This change in conception is best described with an example, such as the microbiome of any living body (including soil), which is more accurately described as diverse and constantly changing in relation to its internal and external environment rather than closed and singular entity [61]. Open ecologies are characterized by openness, permeability and relations that are neither pre-determined nor assumed to be coherent, harmonious or integrated [61]. In a similar vein, this work led us from a closed conception of disciplines to an open ecology of disciplines in a co-creative research process.

The ideas of open ecologies, ecologies of knowledge and one health highlight the role of humans, and the epistemologies that shape their actions, in an ongoing process of becoming with the non-human. The approaches or epistemologies taken by researchers can shape how their studies affect soil. Our discussions throughout this project highlighted that biotechnology often takes an approach of bioprospecting, or genome mining. Biorespecting is an alternative to bioprospecting that could shape how researchers and technologists take on a responsibility and ethic of care with the sites with which they interact, both during and beyond the research project. In the case of biotechnology, this should extend to returning part of any benefits to the ecosystems, including the communities stewarding the site past and present [62]. Biorespecting acknowledges the knowledge embodied in the soil of a land: humans and non-humans alike have co-created the soil. The knowledge embodied in the DNA does not arise spontaneously.

## 3. Conclusion

We found that a public engagement project provided a rare opportunity for transdisciplinary research. It allowed a diverse set of researchers, school children and the general public to co-create a research project. A key outcome was the transformation of a metaphor commonly used to guide soil DNA studies via discussions throughout the project. Of the varied topics discussed, assemblage theory and actor-network theory proved useful as they offered perspectives which removed disciplinary confines and the anthropocentric worldview. As a result, we reflected on properties of soil that can inspire research: soil as a permeable, open, ecology and soil as the root of ‘one health’ [44,61]. Our results revealed microbiomes of varying diversity and composition across the city of Bristol and led to comparisons with ecologies of knowledge [60].

The knowledge in each of the disciplines brought together during this project and in the school classroom is also open and its health is connected through the sharing of ideas. Interdisciplinary research offers an opportunity for epistemologies to be situated relative to one another and the world [63] and in this research project we had the chance to reflect on ways that each discipline relates to soil. The different disciplinary approaches to research grew into one another and their common subject. The resulting approach, biorespecting, offers a fresh outlook which goes beyond the study, to the care of the soil and the ecosystem through sharing the benefits of any information generated. This shift in perspective from an extractive to a collaborative approach could have far-reaching consequences if extended across the sciences.

Epistemologies influence how people study, know and interact with soil, and what soil is and can become—‘what soil is thought to be affects the ways we care for it, and vice versa’ [64]. Our study recognizes the inseparability of nature and culture, since ecologies arise from the relationships between social and biophysical actors, which are best thought of, not as distinct categories, but as the shifting assemblages of an ongoing and always unfinished process of becoming [58].

While scientific research is important for human progress, many global problems arise from human behaviour, which is often guided by metaphor, values and culture [65]. The state of soil is often a consequence of the way that humans relate to soil. We hope that this research helps illustrate how by changing the ideas that shape our actions, we have the chance to rebuild this relationship into one that is both sustainable and beneficial to all.

## 4. Methods

### 4.1. Public engagement activities

The project was designed to engage with people across socioeconomic and age spectrums via events across the city of Bristol in the UK, including the ‘Festival of Nature’ and ‘Fun Palaces’ events, and through several school visits (electronic supplementary material, table S5). The schools that we visited were selected based on their high levels of free school meals (electronic supplementary material, table S4), a proxy for economic deprivation as advised by the Royal Society Public Engagement Team. One activity involved putting on a cape to ‘become’ the sequencing nanopore, taking on the challenge of reading and decoding hidden messages in DNA molecules by ‘feeling’ the unique shape of each nucleotide as it passed through their hands. Using the DNA sequence, they personally decoded, participants then acted as ‘the cell’, translating the mRNA sequence into protein, which took the physical form of making a bracelet. A graffiti wall with prompts offered a chance for participants to share where they would like to carry out sequencing or organisms they would like to sample. To bring the DNA sequencing aspect to life, we used the handheld MinION nanopore sequencer to perform live sequencing demos at these events where participants could see DNA sequences being read by the sequencer in real-time.

### 4.2. Public engagement post-activity questionnaire

The post-activity questionnaire contained three multiple-choice questions to provide feedback regarding each participant's change in understanding and opinion around the approach to research and use of bioengineering. The questions and possible answers (with the number of responses for each answer shown in curly braces) were: Q1. *Having taken part in the activity, how has your understanding of bioengineering changed?* A1.1: I'm more confused than when I started {1}, A1.2: My understanding is similar to when I started {8}, A1.3 I have a better understanding than when I started {49}; Q2. *Having taken part in the activity, are there application areas where you feel it is acceptable for bioengineering to be widely used (select as many as appropriate)?* A2.1: Medicine (e.g. cancer therapies, new forms of drug delivery) {41}, A2.2: Environmental (e.g. pest control, or environmental cleanup) {22}, A2.3 Food (e.g. engineered plants and artificial meat) {10}, A2.4 Materials (e.g. sustainable construction) {41}; Q3. *Having taken part in the activity, how do you feel bioengineering research in general should proceed?* A3.1: I'm not bothered {2}, A3.2: Current processes are fine, nothing needs to change {20}, A3.3 Current processes are mostly fine, but closer links between public and scientists would be helpful for new applications {26}, A3.4 More regulation and oversight is needed because the risks are too high {10}.

### 4.3. High-school lessons

In parallel to the public engagement events, we also wanted people to see the scientific process firsthand and consider the ownership and value of the information any sequencing experiments might uncover. We focused on the value of environmental biodiversity using soil as a focal point. We co-created two lessons which guided students (14–16 years old) through sampling soil, performing DNA sequencing of the microbes within it, discussing the results and finally debating the potential wider impact. These lessons were held at two schools in the Bristol city area (Brunel Academy and Merchants Academy). In the first lesson, students selected a sampling location at their school, took samples and then prepared them for DNA sequencing. After sequencing these samples, which were supplemented by other locations across the city (see 'Soil sampling' section below), the second lesson then looked at the results and considered what might happen if a DNA sequence perceived to be of biotechnological value had been found. The students took on the roles of various interested parties including university scientists that initiated the study, a private multi-national company, an environmental group, the local council and a group of worms living in the field. Each party was then asked to put forward what they would do with the newly found technology and explain how the school and local community might be affected by their plans. Impacts ranged from making the sampling site a nature reserve, to providing money to the students in return for turning the school into a new biotechnology hub.

### 4.4. Transdisciplinary research processes

An active dialogue between the researchers was used to reflect on transdisciplinary approaches in soil research. The public engagement activities took place over the course of a year (electronic supplementary material, table S5); however, the dialogue occurred over a period of 4 years and involved discussions arising from the process of planning, undertaking and writing up the public engagement activities. The discussions covered topics in both synthetic biology and the environmental humanities, centring on the status of nature as a resource, along with other values that could be ascribed to nature. These discussions were informed by other projects the researchers were undertaking at the same time and by a responsible research retreat which preceded the project [66].

As the authors of a recent special issue on transdisciplinary research explain, ‘the global challenges facing us now and in the future demand an urgent and rigorous reassessment of how we conceptualize disciplinary boundaries and the production of knowledge’ [67]. The production and use of antibiotics derived from digital sequence information is one such challenge and we focused on this in the second school lesson (electronic supplementary material, note S2). Though essential for human health and wellbeing, this area of research necessitates questions of the status of nature as a resource, who benefits and who is marginalized in the development of antibiotics, and the relationships between citizens, state and corporate power and non-human life in their production.

The authors of the transdisciplinary special issue also note ‘the lack of focus on how scholars both think about and practice this work across and between the humanities and the sciences' in the literature on transdisciplinarity [68]. There are essentially two sets of challenges to address—the challenges presented by the use and production of antibiotics, and the challenges of developing ‘new modes of collaboration necessary to address them.’ The term ‘biorespecting’ is our attempt to suggest a ‘mutual framing’ of challenges in antibiotics research that can help to bridge disciplinary boundaries and open space for further work, based on collaborations between researchers from the humanities and the sciences, with the necessary transdisciplinary expertise to account for the needs of the many people and beings on whom the search for and production of antibiotics bears [69].

### 4.5. Soil sampling

Samples were collected from six sites in the Bristol metropolitan area: 1. Brunel Academy school (51.468337, –2.531101), 2. Merchant's Academy school (51.409633, –2.611575), 3. Ashton court deer park (51.448750, –2.641000), 4. Queen square (51.450778, –2.594806), 5. Fenswood farm (51.423389, –2.671750) and 6. Avon river bank (51.447917, –2.625278). Using the soilscapes dataset (https://www.landis.org.uk/soilscapes), these sites were classified as the following soil types: 1. Slowly permeable seasonally wet acid loamy and clayey soils, 2. Lime-rich loamy and clayey soils with impeded drainage, 3. Freely draining slightly acid but base-rich soils (texture: loamy), 4. Loamy and clayey floodplain soils with naturally high groundwater, 5. Slowly permeable seasonally wet slightly acid but base-rich loamy and clayey soils; site 6 was on the border of the classifications assigned to (3) and (4). Two samples per site were taken. Soil surface samples (10–20 cm depth) were collected with a 38 mm diameter soil corer (Geopacks) and stored at 0°C for up to 4 h, then at –80°C. At each site, the replicate samples were collected within a 10 m^2^ area. The soil corer was washed with sterile water between sites. These samples were also used as a basis for the live sequencing demonstrations held at local events.

### 4.6. Geochemical assays

All geochemical assays were carried out by the School of Geographical Sciences Laboratory Platform at the University of Bristol, UK. Specifically, measurements of pH (1 g soil in 9 ml milli-Q water), total carbon and total nitrogen by elemental analysis (Vario Pyro Cube EA), particle size distribution (Mastersizer 3000) and organic matter by loss on ignition were performed with approximately 10 g of each soil sample.

### 4.7. Metagenomics DNA extraction and sequencing

We used a metagenomics approach to assess the microbial composition in our soil samples. This is a process in which a particular DNA region is amplified using the polymerase-chain-reaction (PCR). By sequencing this PCR amplified DNA and comparing it to a database of known microbial sequences for that region (which act as barcodes), the microorganisms living in each soil sample could be discerned. We used a universal 16S region as our barcode, which aims to target prokaryotes [70]. This metagenomics approach has several limitations: it does not study all organisms in a soil (e.g. it ignores fungi), bias can arise, and there is no way of knowing whether the analysed DNA arises from living or dead organisms [71–74]. Nonetheless, it offers a snapshot into the microbial composition that could be used to assess major differences between sampling locations and potential impacts of human activity.

DNA extraction from the soil samples was carried out using the Qiagen DNeasy PowerLyzer PowerSoil kit with 0.2–0.3 g of soil thawed for 1 h. A PCR was completed by combining extracted DNA (100 ng) with 10 µM primers (2.5 µl), 10 mM dNTPs (1 µl, NEB N0447S), 10 µl Phusion HF buffer (5X, NEB B0518S) and Phusion DNA polymerase (1 unit, NEB M0530S). 16S PCR primers 27F (AGRGTTYGATYMTGGCTCAG) and 1492R (TACCTTGTTAYGACTT) ordered from Integrated DNA Technologies were used for the PCR [75]. The following PCR reaction was used: initial denaturation (98°C, 30 s), 35 cycles of denaturation (98°C, 7 s), annealing (51°C, 30 s) and extension (72°C, 45 s) followed by a final extension (72°C, 10 min). Samples were purified using Monarch PCR & DNA cleanup kit (NEB T1030S), with elution in nuclease-free water (20 µl). PCR amplicons were checked by gel electrophoresis (1 h, 80 V, 1% agarose, Gel Green dye) by comparison to NEB 1 kb ladder (NEB N3232S). Sequencing kits SQK-LSK108 and EXP-NBD103 (Oxford Nanopore Technologies) were used to prepare the DNA sequencing library, with DNA from each sample (350 ng). The following EXP-NBD103 barcode to sample mapping was used: BC01, BC02 = Brunel Academy school; BC03, BC04 = Ashton court deer park; BC05, BC06 = Fenswood farm; BC07, BC08 = Merchants' Academy school; BC09, BC10 = Queen square; BC11, BC12 = Avon river bank. The library was loaded onto a FLO-MIN106 flow cell and run on a MinION sequencer for 48 h.

### 4.8. Data analysis

The raw sequencing data were basecalled with guppy v.3.2.4 using configuration file ‘dna_r9.4.1_450bps_hac.cfg’ and taxonomic classification was completed using the EPI2ME WIMP (What's In My Pot) workflow [76]. Sample sequencing specifics and base quality were investigated using Nanoplot v.1.41.0 [77]. Soil sample statistical analyses were performed in the R environment v.4.0.2, using R package vegan v.2.5-7 [78]. Cluster analysis was performed with the ‘ward.D2’ method using Euclidean distances of Hellinger-transformed microbial communities at species-level [79]. Microbial richness (e.g. number of species) was performed on the rarefied dataset, *n* = 64 089 which was the number of sequences present in the smallest sample, using vegan v.2.5.6 [78]. Venn diagram was created with ggVennDiagram v.1.1.5 [80]. Principal coordinate analysis (PCoA) was carried out on the Bray–Curtis dissimilarity matrix calculated from Hellinger-transformed species abundance data, using vegan v.2.5.6 and ape v.5.7.1 [81]. The function envfit() from vegan was used to calculate multiple regression of geochemical variables with PcoA axes. Only geochemical variables that showed a significant (*p* < 0.05) correlation with PcoA axes were plotted in the PcoA plot. Mantel tests were performed between the Bray–Curtis dissimilarity matrix calculated from Hellinger-transformed species abundance data, and the Euclidean similarity matrix calculated on the geochemical dataset or the sample geographical distances. Bray–Curtis and Euclidean distances were calculated using the vegan function vegdist(), whereas geographical distances were calculated using distm() with the ‘distHaversine’ method (‘Haversine’ great circle distance), using the library geosphere v.1.5-18. Mantel tests were calculated using Spearman's correlations using the mantel() function from vegan.

## Data Availability

The sequencing data and geochemical analysis generated by this study have been deposited in the OSF database at: https://doi.org/10.17605/OSF.IO/62Q9D [82]. Supplementary material is available online [83].
